# Catwalk: identifying closely related sequences in large microbial sequence databases

**DOI:** 10.1099/mgen.0.000850

**Published:** 2022-06-30

**Authors:** Denis Volk, Fan Yang-Turner, Xavier Didelot, Derrick W. Crook, David Wyllie

**Affiliations:** ^1^​ Nuffield Department of Medicine, University of Oxford, Oxford, UK; ^2^​ School of Life Sciences, University of Warwick, Coventry CV4 7AL, UK; ^3^​ Department of Statistics, University of Warwick, Coventry CV4 7AL, UK; ^4^​ UK Health Security Agency, Forvie Site, Addenbrookes’ Campus, Robinson Way, Cambridge CB2 0SR, UK; ^†^​ Present address: UKRI Science and Technologies Facilities Council, Harwell, UK

**Keywords:** bacterial genomics, microbial relatedness, outbreak detection

## Abstract

There is a need to identify microbial sequences that may form part of transmission chains, or that may represent importations across national boundaries, amidst large numbers of SARS-CoV-2 and other bacterial or viral sequences. Reference-based compression is a sequence analysis technique that allows both a compact storage of sequence data and comparisons between sequences. Published implementations of the approach are being challenged by the large sample collections now being generated. Our aim was to develop a fast software detecting highly similar sequences in large collections of microbial genomes, including millions of SARS-CoV-2 genomes. To do so, we developed Catwalk, a tool that bypasses bottlenecks in the generation, comparison and in-memory storage of microbial genomes generated by reference mapping. It is a compiled solution, coded in Nim to increase performance. It can be accessed via command line, rest api or web server interfaces. We tested Catwalk using both SARS-CoV-2 and *Mycobacterium tuberculosis* genomes generated by prospective public-health sequencing programmes. Pairwise sequence comparisons, using clinically relevant similarity cut-offs, took about 0.39 and 0.66 μs, respectively; in 1 s, between 1 and 2 million sequences can be searched. Catwalk operates about 1700 times faster than, and uses about 8 % of the RAM of, a Python reference-based compression and comparison tool in current use for outbreak detection. Catwalk can rapidly identify close relatives of a SARS-CoV-2 or *M. tuberculosis* genome amidst millions of samples.

## Data Summary

1. Data used for benchmarking:

*Mycobacterium tuberculosis* data was from https://ora.ox.ac.uk/objects/uuid:82ce6500-fa71-496a-8ba5-ba822b6cbb50.

SARS-CoV-2 data was obtained from https://cog-uk.s3.climb.ac.uk/phylogenetics/latest/cog_unmasked_alignment.fasta on 1/12/2021. This file is approximately 60 GB. A 1 % random downsample of this is included in https://github.com/dvolk/catwalk.

The simulated dataset is available at https://github.com/dvolk/catwalk/tree/master/benchmark.

2. Software availability:

the code version submitted for review is v.1.0.0, available as https://github.com/dvolk/catwalk/archive/refs/tags/1.0.0.tar.gz

**Impact Statement**

There is a need for fast computational tools to identify similar microbial genomes, since such tools can detect recent transmissions from within databases of sequenced microbes. However, the huge numbers of viral sequences generated during the SARS-CoV-2 pandemic have challenged existing approaches for doing so. In this work, we present a software tool, Catwalk, which performs such comparisons. It uses various optimizations to speed up comparisons and to reduce the memory usage required; it can compare a SARS-CoV-2 genome with over 2 million others in under a second using standard computational hardware. This is much faster than previous approaches. The tool described will assist public-health programmes in identifying potential transmission events using large sequence databases derived from population level pathogen sequencing.

## Introduction

The last few years have highlighted both the potential and the challenges in guiding public-health action using large-scale microbiological genomics. Sequencing of SARS-CoV-2 genomes on an unprecedented scale has allowed us to identify the diversification, geographical structuring and ongoing transmission of SARS-CoV-2 globally, and has delineated transmission routes locally, nationally and internationally [1, 2]. At the same time, developments in *Mycobacterium tuberculosis* genomics [3] have highlighted the key role of genomic prediction of antibiotic resistance, something that is likely to accelerate use of *M. tuberculosis* genomics for clinical use, thereby providing dense sampling which, at least in developed economies, has already been used successfully to track outbreaks [4].

For both the pathogens above and many others, there is a public-health need to rapidly find sequence pairs compatible with recent transmission. Such an ability underpins rapid detection of *M. tuberculosis* transmission [4], as well as identifying the SARS-CoV-2 importation events that disseminated successful clones widely [1, 2]. In both cases, although indels do evolve and may have important functional implications, single nucleotide variation occurs faster, and is the preferred type of variation from which to undertake fine-scale reconstruction of recent molecular evolution [5–7].

Here, we focus on a generic approach applicable to novel and existing epidemic pathogens where mapping to a reference genome is available. This approach, which relies on storage of the sequence in the form of differences from a reference sequence (reference-based compression), has been previously described [8] and is used by the UK Health Security Agency (UKHSA) for tracking *M. tuberculosis* relatedness [8]. The advantage of this approach is that is does not rely on additional pathogen-specific information, such as the tree required for phylogenetic placement [9], or a core-genome multilocus sequence typing scheme [10]. The disadvantage is a requirement to compare new samples against all existing ones, which is barrier to scalability.

To diminish the impact of the requirement for one-against-all comparisons, we analysed the comparison of reference-based compressed sequences. We present Catwalk, a high-performance, memory-frugal open-source software that addresses two important performance bottlenecks. We compare its performance with that of the findNeighbour2 Python implementation [8], and illustrate algorithm performance using synthetic, *M. tuberculosis* and SARS-CoV-2 genomes.

## Theory and implementation

### Faster, more memory-efficient sequence compression and comparison

Efficient computation of pairwise single nucleotide variant (SNV) distances between DNA sequences derived from reference mapping can be achieved by first computing the differences relative to the reference sequence, and subsequently deducing the pairwise differences [8]. We analysed the performance of this approach, which was previously implemented in Python. In the previous implementation, differences from the references were represented as sets [8]. We noticed that storing large sets might consume significant amounts of RAM simply because of the requirement to store pointers, which are known as references in Python. Pointers are used to link elements in the hash table data structure used for Python 3 set storage and the linked lists containing the values themselves [11]. Therefore, we explored software systems where lower pointer use might result in RAM savings.

Furthermore, we analysed the set operations themselves. The approach described previously [8] considered a mapped sequence S represented as a series of sets S_A_, S_C_, S_G_, S_T_ containing positions of non-reference A, C, G and T bases, and S_N_ containing the positions of bases that could not be called. Similarly, a second sequence R is represented as sets R_A_, R_C_, R_G_, R_T_, R_N._ The difference *d* between S and R can be written as


$$d=\sum_{b\;\in \left\{A,C,T,G\right\}}\left|\left(S_{b}-R_{N}\right)\wedge \left(R_{b}-S_{N}\right)\right|\;\;\;\;\left(eq.\;1\right)$$

where

A – B denotes the difference between sets A and B, defined as the elements found in A and not found in B;

A ∧ B denotes the symmetric difference of sets A and B, defined as the elements found in either A or B but not both; |X| denotes the number of elements in (cardinality of) X.

Thus, the comparison relies on performing eight set differences, and summing the symmetric differences observed. This is evident because if we were computing only differences from the reference for the base A, we can compute


$$\left|\left(S_{A}-R_{N}\right)\wedge \left(R_{A}-S_{N}\right)\right|\;\left(eq.\;2\right)$$

where $S_{A}-R_{N}$ represents the positions in sequence S that are non-reference A at positions which are called in sequence R, so that *eq*. 2 reflects non-reference A present in R or S, but not both.

Given the above, and since our objective is to find small distances, compatible with recent transmission from case to case, we identified three optimizations:

1. Positions containing unknown nucleotides (S_N_, R_N_; *eq*. 1) are represented as sets of integers while non-reference calls (S_b_, R_b_; *eq*. 1) are represented as an ordered list of integers. The implementation chosen (see below) avoids the need for pointers, reducing memory usage.
2. Symmetric differences between S_b_ and R_b_ are identified with a modified symmetric difference algorithm, which is a modification of the approach taken in the corresponding C++ algorithm [12]. It iterates simultaneously over both the ordered lists of differences S_b_ and R_b_, detecting variants present in only one of the two sets, excluding positions with unknown bases (S_N_, R_N_). This has at worst O(|S_b_|+|R_b_|−1) complexity, assuming lookup of whether an unknown position is present in S_N_ or R_N_ has O(1) complexity, as is typically the case for hash-based lookups. However, the modification exits early if distances greater than the prespecified maximum distance reportable are identified. This is acceptable since the distance can only increase (not decrease) during computation. This is an important time-saving optimization, see below.
3. We implemented the algorithm in Nim (https://nim-lang.org/), generating compiled code.

### Components

We built Catwalk as four components, with independent responsibilities in terms of computation and interfaces, allowing users access via command line, rest api and a graphical web browser interface. These components are:

1. engine – a Nim library that stores sequences in a compressed format relative to the genome reference in memory;
2. web server – a web server that runs the engine and offers a RESTful API for genome comparison;
3. web user interface – a web application that connects to a server and allows users to identify and browse those samples that have low single nucleotide distances to an individual genome sequence;
4. clients – command-line and Python tools that connect to a server via RESTful API.

### Benchmarking

We conducted benchmarking on a bare metal desktop computer equipped with an Intel I7 8700 CPU, a solid-state disc and 40 GB of RAM. We used two real data sets (Table 1) and one simulated one representing *in silico* SARS-CoV-2 evolution. We measured time to load a multifasta file into either Catwalk or a reference Python implementation in the *seqComparer* module of findNeighbour2 [8]. We also measured the time to find the neighbours of a random selection of 100 sequences from within the data set, and assessed the impact of differing single nucleotide variation cut-offs on algorithm performance. Both benchmarks examine the performance of the computational engine (component 1 above); the costs of the web server (component 2 above) processing the request and associated http transport performance were not assessed.

**Table 1.** Data sets used for benchmarking

Data set	Description	Nucleotides excluded from comparisons	No. of sequences	Median distance from reference	Median no. of unknown bases
* M. tuberculosis *	Genomes sequenced by UKHSA, mapped and variation called against NC_000962.2 (4 411 532 nt)	557291	12 832	1119	40 369
SARS-CoV-2	Genomes generated by COG-UK consortium, mapped and variation called against MN908947.3 (29 903 nt)	386	1 529 081	17	10.5

## Results

### Catwalk performance relative to a reference implementation

To assess the impact of the optimizations engineered into Catwalk, we compared Catwalk performance with the Python implementation in the *seqComparer* class in the reference Python implementation findNeighbour2 [8]. As test data, we used a data set of 12 832 *M *. *tuberculosis* genomes produced as part of the UKHSA’s routine prospective sequencing of tuberculosis in England (Table 1). We observed an approximately 40-fold improvement in load speeds, 1750-fold speed-up in comparison rates, with memory usage 12-fold lower than used by the reference implementation (Table 2).

**Table 2.** Comparison of *M. tuberculosis* dataset load speed and comparison speed for Catwalk versus findNeighbour2 *seqComparer*

Implementation	Time to load data [s per sample]	Time to query neighbours with 20 SNV cut-off, per sample queried [median (25th, 75th centiles)] [μs]	Memory usage [MB per sequence]
findNeighbour2 *seqComparer* (Python)	0.31	1 700 (1500, 2100)	1.78
Catwalk	0.0076	0.98 (0.91, 1.04)	0.15
Approximate performance difference	40-fold speed-up	1 750-fold speed-up	12-fold lower memory usage

### Determinants of Catwalk performance

**Fig. 1.**
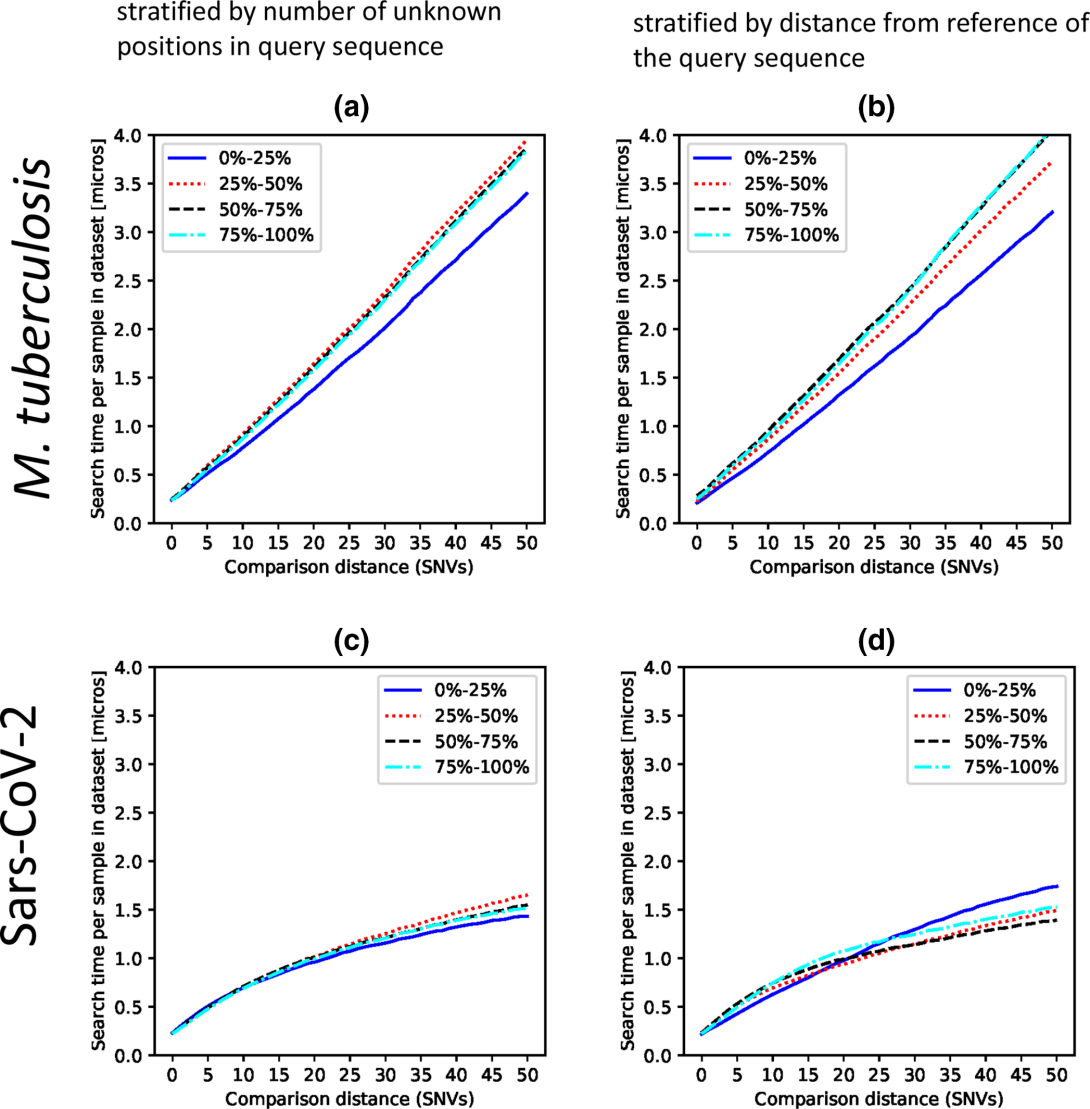
Catwalk search performance. Time taken to search neighbours of a single sample in the *M. tuberculosis* data set (*n*=12 832) (a, b) or a SARS-CoV-2 data set (*n*=1 529 081) (c, d) using different SNV cut-off values. Results are expressed as search time in μs ('micros') per sample in each data set to allow comparisons between data sets. Standard errors of the mean timing are less than 3 % of the plotted values in all cases, and are not shown. In (a) and (c), results are stratified by the number of unknown positions in the sequence used for the query sequence, in quantiles. In (b) and (d), results are stratified by the distance from the reference of the query sequence, in quantiles.

Next, we considered determinants of Catwalk performance. We compared real data sets from *M. tuberculosis* and SARS-CoV-2 (see Table 1). Considering *M. tuberculosis *, search performance is slower as the SNV cut-off used increases (Fig. 1a, b). Searches are also slower if larger numbers of unknown bases are present (Fig. 1a) or if the distance from the reference is larger (Fig. 1b). With a 6 SNV cut-off, which is recommended for use in public-health settings [4], the mean search time per sample in the data set was 0.66 μs.

To further explore the relationship between search times, the number of unknown nucleotides and distance from the reference, we also applied Catwalk to a simulated data set in which genomes are mutated based on the SARS-CoV-2 reference genome. This approach showed similar relationships between distance to reference as was seen in the clinical *M. tuberculosis* data set. The number of unknown bases present in the simulated data (up to 20 % of the genome) had little impact on performance. Results are available in the project GitHub repository.

### Performance of Catwalk in searching UK SARS-CoV-2 genomics data

Finally, we examined performance with a data set of 1.5 million sequences of SARS-CoV-2 generated by the COG-UK consortium (Table 1). The relationship with SNV distances to the reference (Fig. 1c) is similar to that seen with *M. tuberculosis *; the curved shape likely reflects population structuring in the data set used. This is as expected since the virus accrues about 25 mutations per year relative to the ancestral sequence observed in January 2020, with very strong geographical structuring. Additionally, there are less than 25 % unknown bases in the data set, which (as in the simulation) has little impact on search speed (Fig. 1d). With a 3 SNV cut-off, which has been used in nosocomial outbreaks [13], the search time was 0.39 μs per sample in the data set. Thus, for two clinically relevant data sets and SNV cut-offs, the Catwalk tool affords search speeds of more than 1 million samples per second.

## Discussion

The explosion of genome sequence data seen during the SARS-CoV-2 pandemic has exposed the need for new fast tools for sequence comparison. Catwalk is an example of such: applicable to reference-mapped sequence data, it stores reference compressed sequences in RAM, allowing rapid sequence searches, and is optimized for the detection of the short SNV distances that are of public-health relevance. Catwalk is accessible via a rest web services interface, command line or web server interface. Both speed and memory usage are much improved compared to previous Python implementations. Performance is best where samples are close to the reference genome, and with lower numbers of undetermined sites. We observed search times for both SARS-CoV-2 and *M. tuberculosis* genomes produced during public-health sequencing projects of between one and two million samples per second; this represents an approximate 1750-fold speed-up on a previous technology, while also requiring about 8 % of the memory usage of the comparator.

Our tool has several limitations, by design. Firstly, it is applicable to reference-mapped, fixed-length sequence data only. Secondly, it analyses single nucleotide variation, not other classes of genetic variation such as indels. Thirdly, it does not store distances between sequences, although that could be achieved by an external application using Catwalk as a distance computation engine. We do not include this capacity because it can present a significant storage challenge: for the approximately 1.5 million SARS-CoV-2 genomes analysed, there are approximately 9×10^9^ pairs of sequences separated by 0 to 3 SNVs. Storing this amount of data is feasible, but for some applications it may be unnecessary if (as is the case for SARS-CoV-2 genomes) the data can be recomputed faster than it can be loaded out of a database. However, findNeighbour4 [14] is a larger open-source component currently under development that stores Catwalk outputs in a database. Finally, in its current form, Catwalk runs on a single machine only. The algorithm could be refactored to run across a grid of redundant machines, but given the fast load rate, small memory footprint and rapid comparisons achieved this is unnecessary for all applications we attempted, including in SARS-CoV-2.

In summary, we have produced an implementation of a reference-based compression algorithm for fixed-length microbial genomes. This can store sequences and allow pairwise SNV comparisons between millions of SARS-CoV-2 genome pairs. We believe this ultrafast algorithm will find widespread use in public genomics, as it is complementary with existing tools based on phylogenetic placement or core-genome multilocus sequence typing for bacteria.
